# Reemergence of Rabies in Chhukha District, Bhutan, 2008

**DOI:** 10.3201/eid1612.100958

**Published:** 2010-12

**Authors:** Tenzin, Basant Sharma, Navneet K. Dhand, Nilkanta Timsina, Michael P. Ward

**Affiliations:** Author affiliations: The University of Sydney, Camden, New South Wales, Australia (Tenzin, N.K. Dhand, M.P. Ward);; Regional Livestock Development Centre, Gelephu, Bhutan (Tenzin);; Regional Livestock Development Centre, Tshimasham, Bhutan (B. Sharma, N. Timsina)

**Keywords:** Rabies, epidemic, Bhutan, epidemiology, geographic information systems, spatial distribution, viruses, zoonoses, research

## Abstract

From January through July 2008, rabies reemerged in the Chhukha district of southwestern Bhutan. To clarify the distribution and direction of spread of this outbreak, we mapped reported cases and conducted directional tests (mean center and standard deviational ellipse). The outbreak resulted in the death of 97 animals (42 cattle, 52 dogs, and 3 horses). Antirabies vaccine was given free of charge to ≈674 persons suspected to have been exposed. The outbreak spread south to north and appeared to follow road networks, towns, and areas of high human density associated with a large, free-roaming, dog population. The outbreak was controlled by culling free-roaming dogs. To prevent spread into the interior of Bhutan, a well-coordinated national rabies control program should be implemented in disease-endemic areas.

## Abstract 2

TOC summary: A major outbreak affected dogs, domestic livestock, and humans.

Rabies is a fatal zoonosis caused by rabies virus or rabies-related viruses (genus *Lyssavirus*) and transmitted by the bite of a rabid animal (1). Domestic dogs are the main (>95%) source of human rabies infection. An estimated 55,000 persons die of rabies in Asia and Africa each year (2), >20,000 in India alone (3).

In Bhutan, rabies is endemic to the southern districts that border India (4,5). Domestic dogs are the main reservoir and are responsible for spillover infection of other domestic animals, especially cattle. Sporadic human deaths have also been reported in south-central and southwestern rabies-endemic areas of Bhutan (6,7).

On January 23, 2008, a clinical case of rabies in a cow in Dala, a subdistrict of the Chhukha district, was reported and later confirmed by fluorescent antibody test (8,9). The cow had reportedly been bitten ≈3 weeks earlier by a stray dog with suspected rabies. On the same day, another case was reported (and later confirmed by fluorescent antibody test) in a stray dog in the town of Tshimalakha, Bjachho subdistrict. A retrospective epidemiologic field investigation found that an unreported rabies outbreak in dogs had occurred in the southern villages of Dala subdistrict during November and December 2007.

We report a rabies outbreak in the 3 subdistricts of Chhukha district, Bhutan: Dala, Bongo, and Bjachho (Figure 1). To help develop future control programs, our objectives were to 1) describe the spatio–temporal patterns of the outbreak, 2) generate hypotheses about rabies introduction and spread, 3) assess the relationship between animal rabies and public health, and 4) estimate the cost of the outbreak.

**Figure 1:**
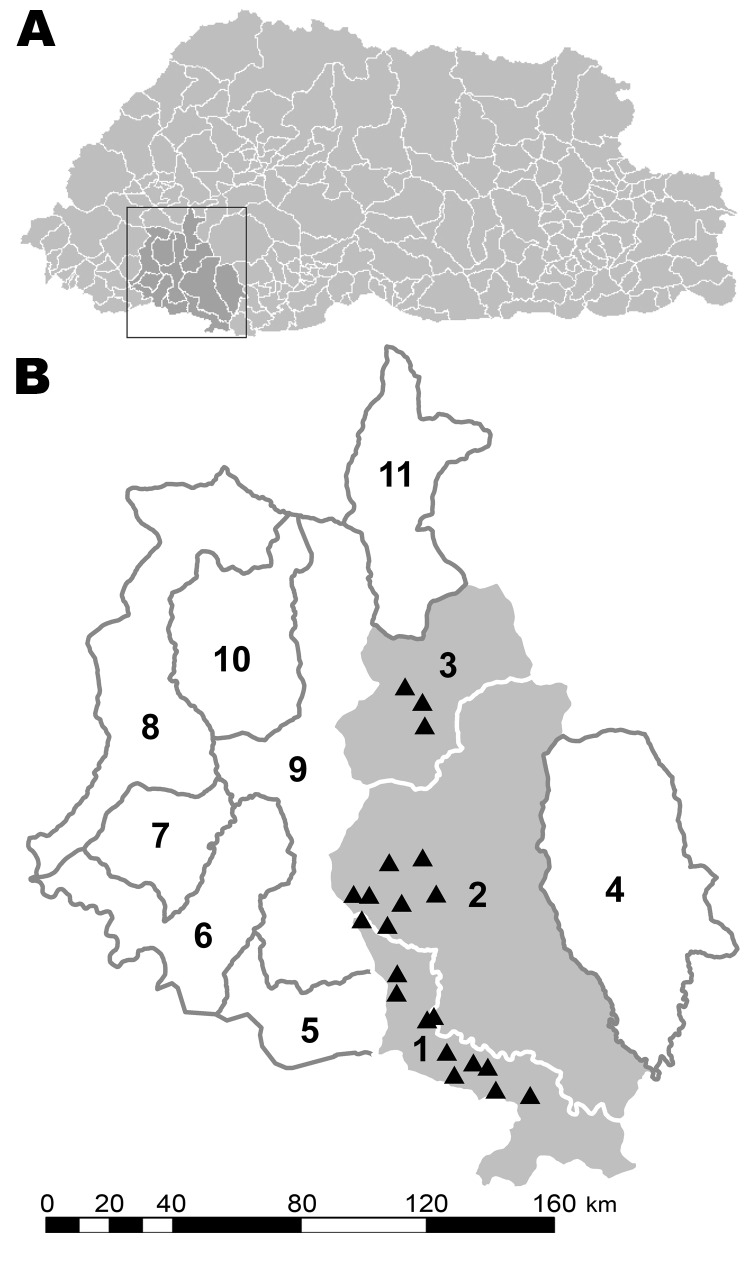
A) Bhutan, with the Chhukha district enclosed. B) The 11 subdistricts of Chukhha district. 1, Dala; 2, Bongo; 3, Bjachho; 4, Genata; 5, Sampheling; 6, Phuentsholing; 7, Logchina; 8, Dungna; 9, Geling; 10, Metap; 11, Chapcha. Gray shading indicates the study areas (1–3); triangles (▲) indicate locations of rabies outbreaks.

## Materials and Methods

### Data Sources

Outbreak data were obtained from the Veterinary Information System database and included case date, number and species of animals affected, location (village X and Y coordinates), subdistrict, and date and type of intervention activities implemented during the outbreak. Data on number of human exposures, reasons, and type and number of postexposure prophylaxis doses administered were acquired from local hospitals. The study was conducted January 23–July 31, 2008.

### Data Analysis

#### Animal Patterns

The attack rate (no. rabies cases/1,000 animals at risk) was calculated for the outbreak period (10). Animal census data for 2008 or dog population data recorded during a vaccination campaign in 2006 in the main towns of Chhukha district were used to calculate attack rates (Table 1) (11). The χ^2^ test was used to compare differences in dog and cattle attack rates among subdistricts. We tabulated the number of persons exposed in each subdistrict, number of persons who received postexposure prophylaxis, and reasons for doing so.

**Table 1:** Attack rates for reported rabies cases, Chhukha district, Bhutan, January 23–July 31, 2008

Subdistrict and species	No. cases/total population	Attack rate (95% confidence interval)
Dala		
Cattle	18/4,194	4 (3–7)
Dogs	12/601	20 (11–34)
Bongo		
Cattle	21/2,898	7 (5–11)
Dogs	32/1,343	24 (17–33)
Bjachho		
Cattle	3/772	4 (1–11)
Dogs	8/707	11 (6–22)

#### Temporal Patterns

The distribution of cases over time was investigated by counting the biweekly number of cases. The relationship between intervention measures (e.g., culling and impounding of free-roaming dogs) and the time series of cases was assessed by counting the number of cases before and after implementation of these control measures.

#### Spatio-Temporal Patterns

The reported cases were mapped (ArcGIS 9.3; ESRI, Redlands, CA, USA) by using a Bhutan shape file (datum: GRS [Geodectic Reference System] 1980, Spheroid; projection: GCS [Geographic Coordinate System] Bhutan Drukref03, Transverse Mercator). The mean center of cases (average X and Y coordinates, useful for tracking changes in distribution) and a standard deviational ellipse (a measure of directional spread), weighted by date of report of cases (12–16), was calculated (Spatial Statistics Tools; ArcGIS 9.3).

### Economics

The cost of the outbreak was analyzed by using 3 simple, direct-cost calculation methods (17). The first calculation was direct cost associated with cattle deaths = number of cattle deaths (n = 42) × average cost of cattle (existing market price of local cattle in Bhutan was Bhutanese ngultrum [Nu.] 10,000). The second calculation was cost of postexposure prophylaxis for humans = total number of human exposures (n = 674) × 5 vaccine doses/person × cost of vaccine (Nu. 450/dose in Bhutan), which was provided free of charge and paid for by the Ministry of Health, Bhutan. The third method was cost of the surveillance and control program, which was calculated on the basis of actual expenditure incurred (removal and impounding of dogs, awareness program), vaccination of ≈200 dogs (at Nu. 25/vaccine dose), and travel and logistics costs for the outbreak response team (paid by the Department of Livestock, Bhutan).

## Results

### Animal Patterns

During the study period, 97 cases of rabies (42 in cattle, 52 in dogs, 3 in horses) were reported in the subdistricts of Dala (18 cattle, 12 dogs), Bongo (21 cattle, 32 dogs and 3 horses), and Bjachho (3 cattle, 8 dogs) (Table 1). Incidence was 5 (95% confidence interval 4–7) and 20 (95% confidence interval 15–26) cases per 1,000 population at risk for cattle and dogs, respectively. Incidence did not differ significantly between the 3 subdistricts for dogs (χ^2^ 3.65, p = 0.16) or cattle (χ^2^ 3.12, p = 0.21) (Table 1).

### Temporal Patterns

The epidemic peak occurred during weeks 11 and 12 (April 3–16), and 65% of cases were reported between weeks 9 and 18 (April and May). The epidemic lasted for 27 weeks and ended in July (Table 2).

**Table 2:** No. rabies cases in animals reported biweekly by subdistrict, Chhukha district, Bhutan, January 23–July 31, 2008

Weeks	DalaV*			Bongo†				Bjaccho‡		Total
	Cattle	Dogs		Cattle	Dogs	Horses		Cattle	Dogs	
1−2	2	00		00	00	00		3	3	8
3−4	1	00		00	00	00		00	00	1
5−6	00	00		00	00	00		00	2	2
7−8	5	00		00	00	00		00	00	5
9−10	1	1		4	3	00		00	1	10
11−12	4	3		6	4	00		00	1	18
13−14	1	2		2	5	00		00	1	11
15−16	00	2		00	9	00		00	00	11
17−18	3	4		00	6	00		00	00	13
19−20	1	00		00	3	00		00	00	4
21−22	00	00		2	00	00		00	00	2
23−24	00	00		3	1	00		00	00	4
25−26	00	00		4	1	2		00	00	7
27	00	00		00	00	1		00	00	1
Total	18	12		21	32	3		3	8	97

*Free-roaming dogs were culled during weeks 6–9 (Mar 27–Feb 27) and week 24 (Jul 3–9). †Free-roaming dogs in Gedu town were culled during weeks 15–20; dogs were impounded during week 17. ‡Dogs in Tshimalakha and Tshimasham towns were impounded during weeks 16 and 17.

### Spatio-Temporal Patterns

**Figure 2:**
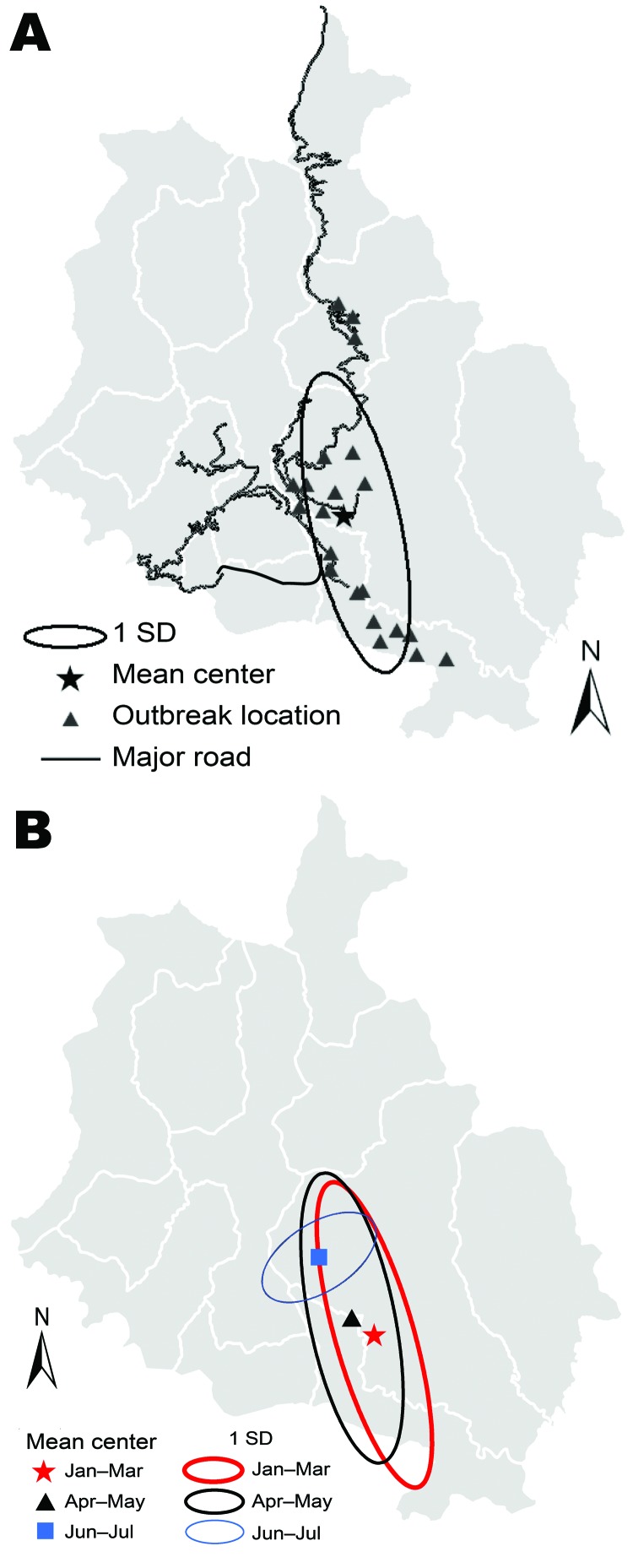
Spatio-temporal patterns of rabies outbreak in the Chhukha district, Bhutan, January 23–July 31, 2008. A) Pattern for the complete outbreak period. B) Patterns during consecutive 2-month intervals during the outbreak period. Jan–Mar period includes January 23–31 (total 69 days; total for other periods 61 days).

The outbreak (mean center X = 2,700,680 meters; Y = 1,014,350 meters) had an ellipsoid (south-to-north) distribution (Figure 2, panel A). The mean center during consecutive 2-month intervals moved northward (Figure 2, panel B). These distributions overlapped and had a south-to-north direction; however, during the final phase (June–July), the outbreak was distributed west to east and spread in the main town areas of Gedu in Bongo subdistrict and its surrounding villages (Figure 2, panel B). The distribution of cases followed the road network and towns with high human density and high numbers of free-roaming dogs (Figure 2, panel A; some road network data not shown).

### Human Exposure Patterns

A total of 674 persons were reported to have been exposed to animals with suspected rabies. Most (77%) exposures were related to contact with rabid animals while either conducting zoosanitary measures or feeding sick animals and by consuming meat and dairy products derived from suspected rabid animals (Table 3). All persons were given antirabies vaccine (5 doses/person) in the hospital. No human deaths were reported during this outbreak.

**Table 3:** No. human exposures to suspected rabies virus, Chhukha district, Bhutan, January 23–July 31, 2008

Subdistrict and exposure	No. persons exposed
Dala	
Contact with rabid animals	30
Bongo	
Dog bite	130
Contact with rabid animals	132
Consumption of meat or dairy products from rabid animals	120
Other animal bite	22
Bjachho	
Consumption of meat from rabid animals	116
Consumption of dairy products from or contact with rabid animals	124
Total	674

### Outbreak Control

In the outbreak areas, free-roaming dogs were culled during weeks 6–9, 15–20, and 24; a total of 500 dogs were impounded during weeks 16 and 17 and remained in shelters until the outbreak subsided. In the adjacent unaffected areas, ≈200 dogs were vaccinated against rabies. The general public and school students in the outbreak areas were made aware, through public meetings and media announcements, of the dangers of rabies. Culling and impounding of free-roaming dogs is believed to have controlled the outbreak; no cases were detected after July (Table 2).

### Outbreak Cost

The direct outbreak cost was estimated to be Nu. 2.75 million (≈US $59,923; 1 US $ = Nu. 46). This cost included cattle deaths (≈Nu. 42,000; 15%); postexposure prophylaxis for humans (≈Nu. 1,516,500; 55%); and implementation of the rabies control program (Nu. 820,000; 30%). The control program cost included ≈Nu. 500,000 for culling, impounding, and awareness programs; ≈Nu. 5,000 for vaccination of domestic dogs; and ≈Nu. 315,000 for the rapid response team (field surveillance and control activities). Because other indirect costs were not taken into account, these costs are likely underestimates.

## Discussion

The rabies outbreak in the Chhukha district initially occurred in dogs in the villages in the southern parts of Dala. The index case dog probably bit several other dogs, resulting in sustained animal-to-animal spread among the free-roaming dog population. After this initial focus of infection, infected free-roaming dogs might have spread the disease by biting cattle and other dogs.

The outbreak spread from south to north and seemed to follow the road network and town areas (Figure 2) that had many free-roaming dogs. High numbers of free-roaming dogs would have provided opportunities for infected dogs to transmit the virus to susceptible dogs and then to cattle in the region. Later, the movement of infected free-roaming dogs from some of these towns might have been responsible for the spread of the disease and spillover infection to cattle in surrounding villages (5). However, the culling of free-roaming dogs possibly removed this rabies reservoir from the outbreak areas, resulting in a drastic reduction in the number of cases by June 2008 (18). This corroborates anecdotal field evidence that immediate removal of reservoirs can facilitate the control of a rabies outbreak. In contrast, in a similar large rabies outbreak in eastern Bhutan from May 5, 2005, through the end of 2007, mass culling was not implemented because of the religious sentiments of the local people (5); in this outbreak, widespread dissemination of rabies persisted for much longer.

Postexposure prophylaxis is crucial for preventing rabies in humans after exposure to any rabid animals. Globally, >10 million persons (mostly in Asia) receive postexposure vaccination against rabies (18,19). In the Chhukha district outbreak, ≈674 persons were given full courses (at 0, 3, 7, 14, and 28 days; Essen regimen) of antirabies vaccine, provided free by hospitals. However, most exposures likely carried low risk, e.g., feeding sick (rabid) cattle, touching carcasses while conducting zoosanitary procedures, and consuming cooked meat and dairy products derived from cattle that had died of rabies (because of lack of knowledge about rabies). Except for the few who handled meat or carcasses or were bitten by dogs, others fell under the World Health Organization exposure category I (touching or feeding animals, licks on intact skin); rabies vaccination is usually not recommended for such exposures (19–22). Probably the fear of rabies sensitized the public and ultimately led to mass vaccination of people. Similar mass vaccination after consumption of dairy products from cattle with suspected rabies or handling of rabid animals and contact with confirmed rabies patients has been reported in Bhutan and elsewhere in the world (5,21–23). There are no specific guidelines to assess such nonbite exposure groups. Should a large-scale exposure occur in the future, use of specific criteria and risk assessment for antirabies vaccination may prevent unnecessary use of scarce vaccine resources, whereas public awareness education might prevent future episodes and potential foodborne transmission (18,21,22). Furthermore, in addition to the existing 5-dose intramuscular Essen regimen followed in Bhutan, other postexposure prophylaxis regimens, such as the intradermal method approved by the World Health Organization Expert Committee, should be reviewed because this method is immunogenic, effective, requires fewer doses of vaccine, and costs 70% less than the conventional intramuscular regimen (19,20,24,25).

The estimated cost of this outbreak was large by Bhutan standards and reflects the extent of rabies in a resource-limited country (2,4,17). Globally, it has been estimated that > US$ 1 billion per year is spent on rabies prevention programs, mostly on postexposure prophylaxis (18,19). Similarly, in the Chhukha district outbreak, 55% of the estimated total costs were associated with postexposure prophylaxis for humans. Although vaccinations were free for the recipients, the cost to the Ministry of Health was high. Because the program to eliminate rabies in dogs contributes to the elimination of rabies in humans (or reduces the cost of postexposure prophylaxis), public health and animal health efforts should emphasize the need for control and elimination of rabies in animal reservoirs. In Bhutan, the resources allocated to rabies control in the animal health sector are inadequate and often lead to low vaccination coverage of dogs. Therefore, financial resources should be shared by the public health sector for effective implementation of rabies control and dog management programs.

In conclusion, the Chhukha district rabies outbreak spread consistently from south to north, following the distribution of roads and towns that had large free-roaming dog populations. Rapid culling of in-contact and unvaccinated free-roaming dogs controlled this outbreak. A similar strategy should be considered for any future rabies outbreaks in Bhutan. Because of the risk for spread of rabies from the southern rabies-endemic zone to the rabies-free interior of Bhutan, a well-coordinated national rabies control program should be implemented to prevent and control rabies in Bhutan.
